# Association between serum fibroblast growth factor‐23 concentration and development of hyperphosphatemia in normophosphatemic dogs with chronic kidney disease

**DOI:** 10.1111/jvim.16237

**Published:** 2021-08-21

**Authors:** Hirosumi Miyakawa, Huai‐Hsun Hsu, Mizuki Ogawa, Ryota Akabane, Yuichi Miyagawa, Naoyuki Takemura

**Affiliations:** ^1^ Laboratory of Veterinary Internal Medicine II Nippon Veterinary and Life Science University Tokyo Japan

**Keywords:** canine, CKD‐MBD, progression, renal

## Abstract

**Background:**

Fibroblast growth factor (FGF)‐23 is increased first in the sequence of changes associated with chronic kidney disease (CKD)‐mineral and bone disorder. Thus, its measurement may serve as a predictive indicator of incident hyperphosphatemia.

**Objectives:**

To investigate whether serum FGF‐23 concentration in normophosphatemic dogs with CKD is associated with the risk of the subsequent development of hyperphosphatemia and CKD progression.

**Animals:**

Forty‐two normophosphatemic dogs with CKD.

**Methods:**

Blood samples and medical records were retrospectively investigated. Hyperphosphatemia was defined as a serum phosphorous concentration >5.0 mg/dL. Progression was defined as a >1.5‐fold increase in serum creatinine concentration. The time periods and hazard ratios for these outcomes were assessed using Kaplan‐Meier analysis, log‐rank test, and univariate Cox regression analysis. The variables associated with the outcomes in the univariate analysis were included in the multivariate Cox regression model with backward selection.

**Results:**

Serum FGF‐23 concentration >528 pg/mL was associated with a shorter time to development of hyperphosphatemia (*P* < .001) and CKD progression (*P* < .001). In multiple Cox regression analysis, increased FGF‐23 concentration remained a significant variable associated with these outcomes (*P* < .05).

**Conclusions and Clinical Importance:**

Increased FGF‐23 concentration in normophosphatemic dogs with CKD was associated with significant risk of development of hyperphosphatemia, independent of CKD stage, and of the progression of CKD. Future research focusing on whether interventions that decrease FGF‐23 secretion will slow the development of hyperphosphatemia and CKD progression is needed.

**Abbreviations**
CI: confidence interval
CKD: chronic kidney disease
FGF: fibroblast growth factor
GFR: glomerular filtration rate
HR: hazard ratio
IRIS: International Renal Interest Society
MBD: mineral and bone disorder
PTH: parathyroid hormone
SBP: systolic blood pressure
UN: urea nitrogen
UPC: urine protein/creatinine ratio
USG: urine specific gravity

## 1 INTRODUCTION

Chronic kidney disease (CKD) is a common, irreversible, and progressive disease in dogs.^1^ In patients with CKD, mineral metabolism disorders such as hyperphosphatemia, renal hyperparathyroidism, and decreased calcitriol synthesis occur because of impaired renal function and are associated with a poor prognosis.^2^, ^3^, ^4^ Therefore, these disorders are considered important complications and are termed CKD—mineral and bone disorder (MBD).^2^, ^3^, ^4^, ^5^ Hyperphosphatemia is known to be a prognostic factor for shorter survival time in dogs with CKD.^6^, ^7^, ^8^, ^9^, ^10^ Therefore, prevention and management of hyperphosphatemia may improve the prognosis of dogs with CKD.

Fibroblast growth factor (FGF)‐23 is a phosphaturic hormone associated with CKD‐MBD.^2^, ^3^, ^5^ Fibroblast growth factor‐23 is released from osteocytes in response to increased serum phosphorus and calcitriol concentrations and promotes phosphate excretion into the urine by downregulation of the sodium‐phosphate co‐transporter in renal proximal tubular cells and inhibition of calcitriol synthesis.^11^, ^12^ Fibroblast growth factor‐23 acts by binding to the FGF receptor‐α‐klotho complex.^13^ In humans, cats, and dogs with CKD, circulating FGF‐23 concentrations have been shown to increase in the advanced CKD stages.^14^, ^15^, ^16^, ^17^, ^18^, ^19^ Increased FGF‐23 concentration in patients with CKD is associated with various mechanisms, such as a decreased clearance of FGF‐23 because of decreasing glomerular filtration rate (GFR), compensation for the accumulation of phosphate in the body, and compensation for decreased klotho protein concentrations.^20^, ^21^, ^22^ Studies of humans and cats with CKD have reported that concentrations of FGF‐23 increased earlier than did those of parathyroid hormone (PTH) and phosphorus,^15^, ^16^, ^17^, ^23^ and were associated with shorter survival time.^24^, ^25^, ^26^ Therefore, FGF‐23 has been noted as an early marker of CKD‐MBD.^2^ Similarly, blood FGF‐23 concentrations in dogs with CKD have been shown to become increased earlier than serum phosphorous concentrations.^18^, ^19^ Therefore, increased blood FGF‐23 concentrations plausibly can reflect phosphate accumulation in dogs with CKD but without overt hyperphosphatemia. Thus, we hypothesized that FGF‐23 predicts development of hyperphosphatemia in normophosphatemic dogs with CKD. If correct, FGF‐23 may be a useful marker of when to initiate a phosphate‐restricted diet to prevent the development of hyperphosphatemia.

Our purpose was to investigate the relationship between FGF‐23 concentration and subsequent development of hyperphosphatemia and progression of CKD and determine the clinical relevance of increased FGF‐23 concentration in normophosphatemic dogs with CKD.

## 2 MATERIALS AND METHODS

### 2.1 Case selection

Ours was a retrospective study performed using medical records and stored serum samples that were collected between September 2014 and September 2020 from client‐owned dogs diagnosed with CKD at the nephrology service of a veterinary medical teaching hospital in Japan. Dogs with CKD were selected from a database using commercially available software (Microsoft Excel, Microsoft Japan Co., Ltd., Tokyo, Japan) and the search terms “CKD” and “dog.” Then, the medical records and presence or absence of serum samples for the dogs identified from the database were reviewed. If serum samples at the time of CKD diagnosis were unavailable, samples obtained at a subsequent hospital visit were selected for measurement of serum FGF‐23 and intact PTH concentrations. A diagnosis of CKD was based on persistently (≥3 months) having at least 1 of the following criteria: renal azotemia, renal proteinuria, or renal abnormalities on abdominal ultrasonography. Renal azotemia was defined as a serum creatinine concentration >1.45 mg/dL (the upper limit of reference range at our institution) and urine specific gravity (USG) <1.030. Renal proteinuria was assessed based on a urine protein/creatinine ratio (UPC) ≥0.5 without an apparent prerenal or postrenal cause. Ultrasonographic renal abnormalities included small kidneys, decreased corticomedullary differentiation, irregular renal contours, or some combination of these findings. Dogs with CKD were classified according to the 2019 International Renal Interest Society (IRIS) CKD guidelines for staging based on serum creatinine concentrations as stage 1 (<1.4 mg/dL), stage 2 (1.4‐2.8 mg/dL), stage 3 (2.9‐5.0 mg/dL), and stage 4 (>5.0 mg/dL).^27^ All dogs with stage 1 CKD had renal proteinuria, ultrasonographic renal abnormalities, or both.

Dogs suspected or diagnosed with neoplasia, hyperadrenocorticism, hypoadrenocorticism, acute kidney injury or acute CKD exacerbation, or having a history of hyperphosphatemia were excluded from the study. Hyperphosphatemia was defined as a serum phosphorous concentration >5.0 mg/dL, based on the reference interval at our institution. In addition, dogs for which follow‐up information could not be obtained were excluded.

### 2.2 Medical records review and data collection

Information regarding signalment, serum biochemical analysis, blood gas analysis, urinalysis, noninvasively determined systolic blood pressure (SBP) and treatment were collected from the medical records. Serum biochemical analyses were performed using an automated analyzer (7180 Biochemistry Automatic Analyzer, Hitachi High‐Technologies Corp., Tokyo, Japan). Blood ionized calcium and bicarbonate concentrations were measured using a blood gas analyzer (GEM PREMIER 3500, Instruments Laboratory Co., Ltd, Tokyo, Japan). Blood samples for blood gas analysis were collected into heparinized tubes (FUJI HEPARIN TUBE, FUJI FILM Corp., Tokyo, Japan). Urine samples were obtained by voiding, urethral catheterization, or cystocentesis. Urine chemical analyses were performed using an automated analyzer (7180 Biochemistry Automatic Analyzer, Hitachi High‐Technologies Corp.). Blood pressure measurements were assessed using oscillometric methods (BP100D hemomanometer, Fukuda M‐E Kogyo Co., Ltd, Tokyo, Japan), according to the recommendations of the American College of Veterinary Internal Medicine.^28^

### 2.3 FGF‐23 and intact PTH

Blood samples were collected with the informed consent of the dogs' owners. Blood samples in tubes containing serum separators were centrifuged at 1181*g* for 5 minutes, and the obtained serum samples were stored at −30°C for submission to an external laboratory (FUJIFILM VET Systems Co., Ltd, Tokyo, Japan) for FGF‐23 and intact PTH analysis. Serum FGF‐23 concentrations were measured using a sandwich ELISA kit (MedFrontier FGF‐23, Hitachi Chemical Diagnostics Systems Co., Ltd, Tokyo, Japan). Serum intact PTH concentrations were analyzed using a chemiluminescent enzyme immunoassay (Siemens Immulyze intact PTH III, Siemens Healthcare Diagnostics Co., Ltd, Tokyo, Japan). Validation of the FGF‐23 and intact PTH assays was reported in a previous study.^19^

### 2.4 Statistical analysis

Statistical analysis was performed using commercial software (SPSS 24 for Windows, IBM Japan, Ltd, Tokyo, Japan) and open‐access statistical software (EZR software version 1.40, Jichi Medical University Saitama medical Center, Saitama, Japan).^29^ *P* < .05 was considered statistically significant.

The number of days from the day on which the serum sample for FGF‐23 assessment was obtained (baseline) until the development of hyperphosphatemia or progression of CKD was retrieved from the medical records. The frequency of follow‐up (ie, reassessment of serum creatinine and phosphorous concentrations) was determined based on clinical recommendations for individual cases. Development of hyperphosphatemia was defined as serum phosphorous concentration >5.0 mg/dL. Progression of CKD was defined as a 1.5‐fold increase in serum creatinine concentration in comparison with that measured at the time of evaluation of FGF‐23. Dogs were followed until January 2021. Dogs that did not develop hyperphosphatemia did not experience progression at the end of the follow‐up period and those that were lost to follow‐up were censored.

Kaplan‐Meier curves were used to plot the time to development of hyperphosphatemia and CKD progression. The cutoffs for FGF‐23 and intact PTH were based on the reference ranges (528 and 8.5 pg/mL, respectively) reported in a previous study.^19^ The cutoffs for serum biochemical variables other than serum phosphorous concentration were based on the reference ranges at our institution. The cutoff for serum phosphorous concentration (4.6 mg/dL) was set according to the therapeutic aim proposed by the IRIS CKD guidelines.^30^ The cutoffs for bicarbonate and ionized calcium concentrations were determined based on a previous report.^31^, ^32^ Dogs were also divided in terms of USG (≥1.030 and <1.030) and UPC (≥1.0 and <1.0).^32^, ^33^ Systemic hypertension was defined as SBP ≥160 mmHg at baseline.^28^, ^34^ Log‐rank tests were used to compare time to the outcomes across groups. Hazard ratios (HRs) of FGF‐23 and other variables for the outcomes were calculated using univariate Cox regression. Proportional hazard assumptions were analyzed using Schoenfeld residuals. All variables significantly associated with the outcomes in univariate Cox analysis (*P* < .05) were included in the multiple Cox regression model with backward selection. Residual analysis was performed using Martingale residuals.

## 3 RESULTS

### 3.1 Dogs

One‐hundred forty‐eight dogs with CKD were identified from the database. From this group, 45 dogs without serum samples, 11 dogs with neoplasia, hyperadrenocorticism or acute CKD exacerbation, and 24 dogs with hyperphosphatemia or a history of hyperphosphatemia were excluded. In addition, 26 dogs without follow‐up information were excluded. Finally, 42 normophosphatemic dogs with CKD were included in our study. Twenty‐three dogs developed hyperphosphatemia during the follow‐up period and CKD progressed in 18 dogs. They were grouped as stage 1 (n = 23), stage 2 (n = 13), stage 3 (n = 5), and stage 4 (n = 1) CKD (Table 1). Because only 1 dog had stage 4 CKD, it was categorized as stage 3 for statistical analysis. In all dogs with CKD, the median age (range) was 9.3 years (1.4‐15.8 years), and the median body weight (range) was 4.5 kg (1.4‐31.2 kg). The study population consisted of 3 intact males, 16 castrated males, 4 intact females, and 19 spayed females. Breeds included Yorkshire Terrier (n = 11), Papillon (n = 5), Chihuahua (n = 3), Shiba (n = 3), Shih Tzu (n = 3), Toy Poodle (n = 3), Jack Russell Terrier (n = 2), Miniature Schnauzer (n = 2), and 1 of each of the following breeds: Border Collie, Bulldog, French Bulldog, Italian Greyhound, Labrador Retriever, Maltese Miniature, Dachshund, mixed breed dog, Staffordshire Bull Terrier, and Welsh Corgi. Seventeen dogs already were being fed a renal diet at baseline. During the follow‐up period, 9 of the 23 dogs that developed hyperphosphatemia and 6 of the 19 dogs that did not develop hyperphosphatemia were given a renal diet. In addition, 9 of the 18 dogs with CKD progression and 6 of the 24 dogs without CKD progression were given a renal diet during the follow‐up period. No significant difference was found in the interval for the development of hyperphosphatemia and the progression of CKD between dogs with and without renal dietary treatment during the follow‐up period (*P* = .77 and *P* = .57, respectively). No dog received a phosphorous binder during the follow‐up period. Of the 42 dogs with CKD, 25 had been treated with renin‐angiotensin system inhibitors to manage proteinuria or systemic hypertension. More specifically, they were treated with telmisartan (n = 18), benazepril hydrochloride (n = 2), enalapril maleate (n = 1), temocapril hydrochloride (n = 1), and a combination of telmisartan and benazepril hydrochloride (n = 3). No dog had a urinary tract infection based on microscopic examination of urine sediment. The median time (range) between re‐examination visits was 56 days (13‐99 days) in dogs that developed hyperphosphatemia and 91 days (34‐882 days) in those that did not develop hyperphosphatemia. The median time (range) between follow‐up visits was 294 days (15‐259 days) in dogs with CKD progression and 82 days (13‐882 days) in those without CKD progression.

**TABLE 1:** Median (range) values for characteristics of dogs with CKD included in the present study

	Stage 1 (n = 23)		Stage 2 (n = 13)		Stage 3–4 (n = 6)	
Variables	Median (range)	n	Median (range)	n	Median (range)	n
Age (years)	9.4 (3.0‐15.8)	23	9.2 (2.2‐13.5)	13	11.3 (1.4‐14.8)	6
Body weight (kg)	4.0 (1.4‐21.2)	22	6.2 (1.7‐31.2)	13	4.9 (2.4‐14.3)	6
FGF‐23 (pg/mL)	442 (172‐1545)	23	843 (281‐1314)	13	1378 (605‐1754)	6
Intact PTH (pg/mL)	6.5 (5.9‐16.5)	23	6.6 (6.1‐34.6)	13	13.4 (6.1‐24.4)	5
Urea nitrogen (mg/dL)	38.8 (7.1‐66.7)	23	32.3 (17.1‐79.3)	13	72.2 (44.7‐95.6)	6
Creatinine (mg/dL)	0.9 (0.6‐1.39)	23	1.85 (1.45‐2.36)	13	4.01 (3.22‐5.82)	6
Total protein (g/dL)	5.9 (4.8‐7.3)	23	6.0 (4.1‐7.6)	13	6.0 (5.6‐6.8)	6
Albumin (g/dL)	2.4 (1.8‐3.4)	23	2.6 (1.6‐3.5)	13	2.7 (2.4‐3.3)	6
Total calcium (mg/dL)	10.0 (8.0‐12.0)	23	10.2 (8.8‐11.8)	13	11.1 (9.8‐12.6)	6
Phosphorus (mmol/L)	3.6 (1.6‐4.9)	23	3.6 (2.2‐4.6)	13	3.5 (2.7‐4.0)	6
Potassium (mEq/L)	4.6 (3.6‐5.6)	23	4.8 (4.1‐5.9)	12	4.8 (3.7‐5.5)	6
Ionized calcium (mmol/L)	1.22 (0.92‐1.34)	18	1.22 (1.14‐1.28)	12	1.21 (1.14‐1.26)	6
HCO_3_^−^ (mmol/L)	22.3 (17.1‐29.1)	18	23.6 (11.6‐26.6)	12	21.4 (18.3‐24.3)	6
Urine specific gravity	1.021 (1.004‐1.050)	21	1.019 (1.004‐1.023)	13	1.011 (1.009‐1.014)	6
UPC (g/gCre)	2.5 (0.07‐9.3)	22	2.2 (0.04‐12.1)	11	0.12 (0.03‐0.56)	6
SBP (mmHg)	152 (104‐191)	17	148 (123‐235)	9	145 (119‐172)	4

Abbreviations: CKD, chronic kidney disease; FGF, fibroblast growth factor; PTH, parathyroid hormone; SBP, systolic blood pressure; UPC, urine protein : creatinine ratio.

### 3.2 Relationship between FGF‐23 and development of hyperphosphatemia

**FIGURE 1:**
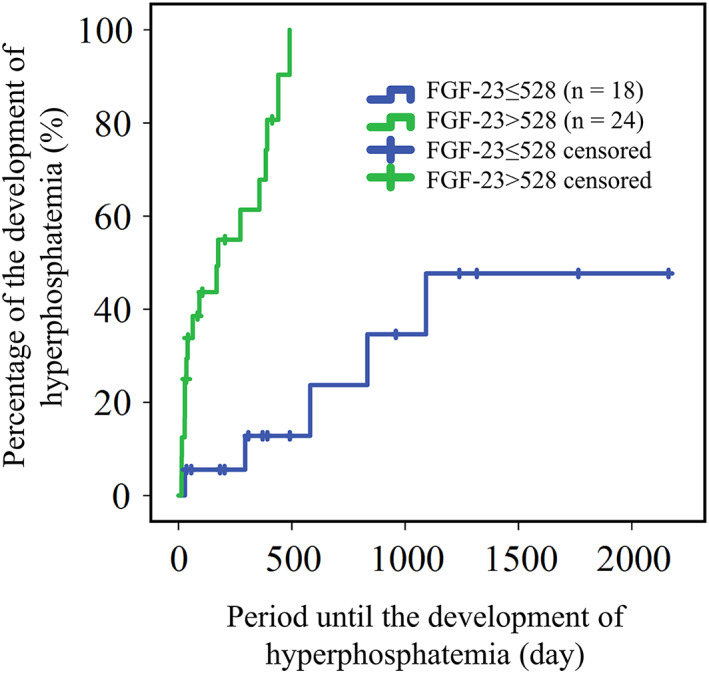
Kaplan‐Meier curve for the time period to the development of hyperphosphatemia in normophosphatemic chronic kidney disease dogs with serum fibroblast growth factor (FGF)‐23 concentrations ≤528 pg/mL (blue line) and >528 pg/mL (green line)

**FIGURE 2:**
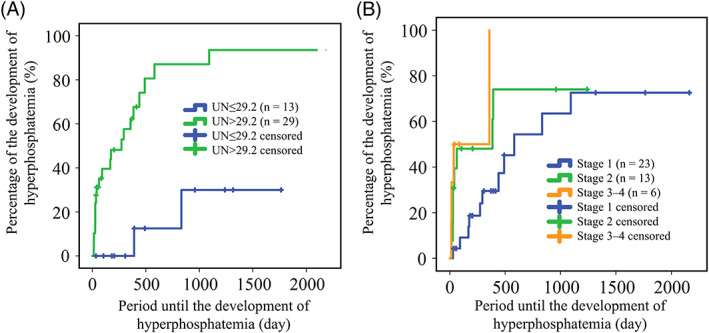
Kaplan‐Meier curve for the time period to the development of hyperphosphatemia in normophosphatemic chronic kidney disease (CKD) dogs with (A) serum urea nitrogen (UN) concentrations ≤29.2 mg/dL (blue line) and >29.2 mg/dL (green line); (B) stage 1 (blue line), stage 2 (green line), and stages 3‐4 (orange line) CKD

The time to development of hyperphosphatemia in dogs with CKD having FGF‐23 concentrations >528 pg/mL (median time, 175 days; 95% confidence interval [CI], 0‐395 days) was significantly shorter than that of dogs with FGF‐23 concentrations ≤528 pg/mL (median time was not reached during the study period, *P* < .001; Figure 1). Serum urea nitrogen (UN) concentration >29.2 mg/dL (median time, 273 days; 95% CI, 93‐453 days) was significantly associated with shorter interval until the development of hyperphosphatemia in comparison with UN ≤29.3 mg/dL (median time was not reached during the study period; *P* < .001; Figure 2A). International Renal Interest Society stage also was significantly associated with the interval to outcome. Median durations (95% CI) to the outcome in stages 1, 2, and 3‐4 groups were 581 days (185‐977 days), 385 days (0‐871 days), and 35 days (0‐240 days), respectively (Figure 2B).

The univariate Cox regression analysis showed that FGF‐23 >528 pg/mL, UN >29.2 mg/dL, and CKD stages 3‐4 were significantly associated with risk for development of hyperphosphatemia (*P* < .001, *P* = .003, and *P* = .01, respectively; Table 2). In addition, time between re‐examination visits correlated with significant risk for outcome (HR, 0.92; 95% CI, 0.89‐0.96; *P* < .001). When these variables were included in the multivariate Cox regression analysis, FGF‐23 >528 pg/mL (HR, 5.66; 95% CI, 1.24‐25.81; *P* = .03), UN >29.2 mg/dL (HR, 6.31; 95% CI, 1.20‐33.10; *P* = .03), and time between re‐examination visits (HR, 0.91; 95% CI, 0.87‐0.95; *P* < .001) were significantly associated with risk of hyperphosphatemia (Table 3).

**TABLE 2:** Hazard ratios for the development of hyperphosphatemia and CKD progression analyzed using univariate Cox regression model in normophosphatemic dogs with CKD

Variables	Cutoff		Development of hyperphosphatemia			Progression of CKD		
		n	Hazard ratio	95% Confidence interval	*P* value	Hazard ratio	95% Confidence interval	*P* value
FGF‐23 (pg/mL)	≤528	18						
	>528	24	15.39	3.41‐69.57	<.001	8.98	2.38‐33.89	.001
Intact PTH (pg/mL)	≤ 8.5	30						
	>8.5	11	2.02	0.80‐5.12	.14	2.21	0.78‐6.24	.14
Urea nitrogen (mg/dL)	≤29.2	13						
	>29.2	29	9.44	2.18‐40.89	.003	3.97	1.14‐13.87	.03
Creatinine (mg/dL)	≤1.45	24						
	>1.45	18	2.23	0.97‐5.12	.06	2.14	0.83‐5.49	.12
Total protein (g/dL)	≥4.9	39						
	<4.9	3	1.41	0.42‐4.80	.58	1.92	0.55‐6.71	.31
Albumin (g/dL)	≥2.0	34						
	<2.0	8	2.06	0.84‐5.03	.11	1.8	0.67‐4.88	.25
Total calcium (mg/dL)	≥9.1	38						
	<9.1	4	1.11	0.33‐3.79	.87	0.79	0.18‐3.51	.75
Phosphorus (mg/dL)	≤4.5	38						
	>4.5	4	1.94	0.65‐5.80	.23	1.04	0.24‐4.58	.96
Potassium (mEq/L)	≤5.1	38						
	>5.1	3	2.34	0.53‐10.30	.26	5.02	1.06‐23.87	.04
Bicarbonate (mmol/L)	>18.8	31						
	≤18.8	5	1.49	0.53‐4.19	.45	1.88	0.64‐5.48	.25
Ionized calcium (mmol/L)	>1.15	27						
	≤1.15	9	0.96	0.37‐2.48	.93	0.74	0.24‐2.32	.61
USG	≥1.030	5						
	<1.030	35	2.22	0.52‐9.56	.28	1.52	0.35‐6.64	.58
UPC (g/gCre)	<1.0	14						
	≥1.0	25	1.18	0.46‐3.04	.74	1.19	0.38‐3.71	.76
SBP (mmHg)	<160	19						
	≥160	11	1.28	0.43‐3.84	.66	0.86	0.23‐3.20	.82
Renal diet during the follow‐up period	Not performed	27						
	performed	15	0.88	0.38‐2.05	.77	1.31	0.52‐3.34	.57
CKD stage	Stage 1	23						
	Stage 2	13	2.10	0.84‐5.27	.11	2.02	0.71‐5.1	.19
	Stages 3‐4	6	4.87	1.43‐16.6	.01	12.41	2.47‐72.97	.002

Abbreviations: CKD, chronic kidney disease; FGF, fibroblast growth factor; PTH, parathyroid hormone; SBP, systolic blood pressure; UPC, urine protein creatinine ratio; USG, urine specific gravity.

**TABLE 3:** Hazard ratios for the development of hyperphosphatemia and CKD progression analyzed using multivariate Cox regression model in normophosphatemic dogs with CKD

Variables	Hazard ratio	95% Confidence interval	*P* value
Development of hyperphosphatemia			
FGF‐23 >528 pg/mL	5.66	1.24‐25.81	.03
Urea nitrogen >29.2 mg/dL	6.31	1.20‐33.10	.03
Time between re‐examination visits (day)	0.91	0.87‐0.95	<.001
Progression of CKD			
FGF‐23 >528 pg/mL	11.33	2.85‐44.98	<.001
Potassium >5.1 mEq/L	12.74	2.12‐76.73	.005

Abbreviations: CKD, chronic kidney disease; FGF, fibroblast growth factor.

### 3.3 Relationship between FGF‐23 and progression of CKD

**FIGURE 3:**
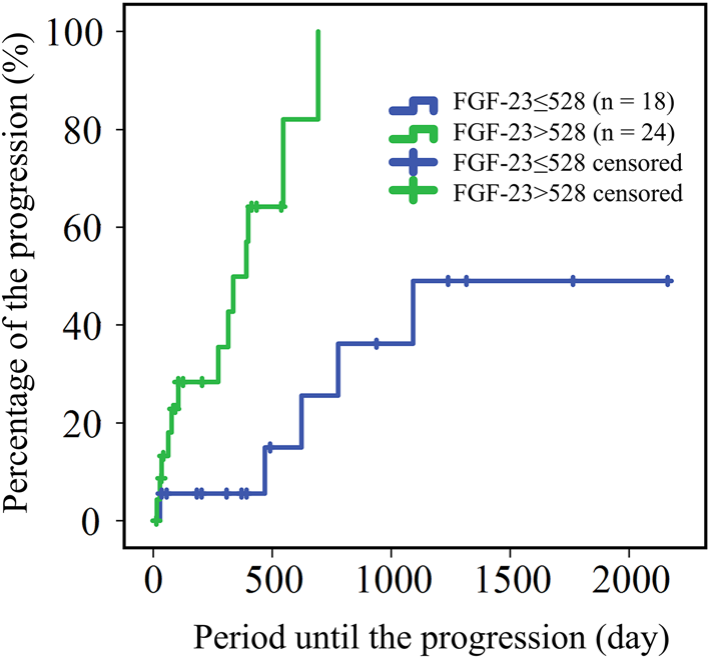
Kaplan‐Meier curve for the time period to the progression of chronic kidney disease in dogs without hyperphosphatemia with serum fibroblast growth factor (FGF)‐23 concentrations ≤528 pg/mL (blue line) and >528 pg/mL (green line)

**FIGURE 4:**
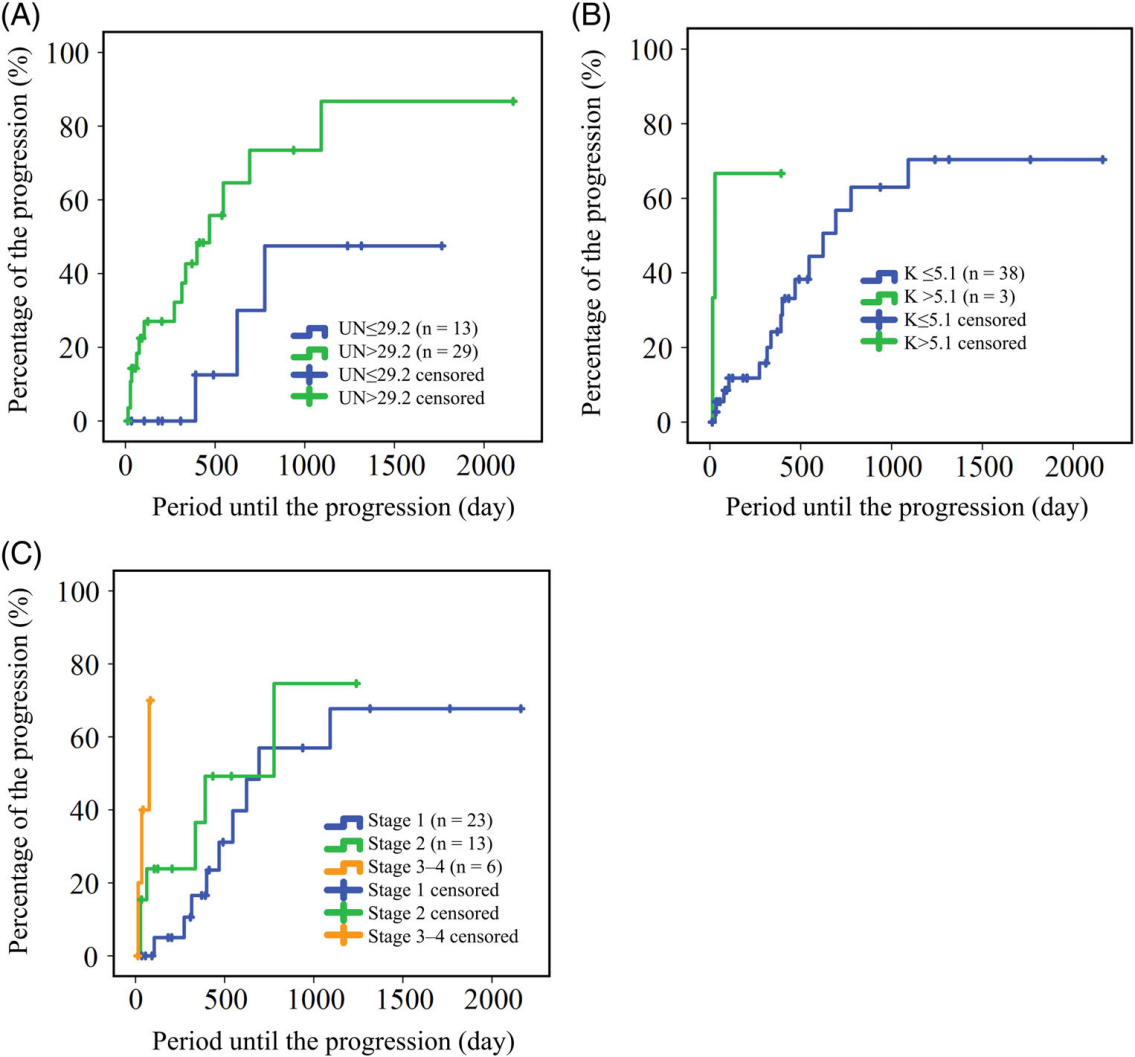
Kaplan‐Meier curve for the time period to the progression of chronic kidney disease (CKD) in dogs without hyperphosphatemia with, A, serum urea nitrogen (UN) concentrations ≤29.2 mg/dL (blue line) and >29.2 mg/dL (green line); B, serum potassium (K) concentrations ≤5.1 mEq/L (blue line) and >5.1 mEq/L (green line); and, C, stage 1 (blue line), stage 2 (green line), and stages 3‐4 (orange line) CKD

Time to progression of CKD in dogs with FGF‐23 concentrations >528 pg/mL (median time, 391 days; 95% CI, 67‐522 days) was significantly shorter than that of dogs with FGF‐23 concentrations ≤528 pg/mL (median time was not reached during the study period; *P* < .001; Figure 3). In addition, serum UN concentration >29.2 mg/dL, serum potassium concentration >5.1 mEq/L, and CKD stage were significantly associated with time to outcome (*P* = .03, *P* = .02, and *P* = .001, respectively; Figure 4). The median duration (95% CI) to CKD progression in dogs with UN >29.2 mg/dL was 469 days (118‐700 days), but the median duration with UN ≤29.2 mg/dL group was not reached during the study period. Median durations (95% CI) to CKD progression for serum potassium concentrations >5.1 and ≤5.1 mEq/L groups were 28 days (7‐49 days) and 623 days (363‐833 days), respectively, whereas those in the CKD stages 1, 2, and 3‐4 groups were 693 days (460‐926 days), 777 days (328‐1227 days), and 77 days (12‐143 days), respectively.

The univariate Cox regression analysis showed that FGF‐23 >528 pg/mL, UN >29.2 mg/dL, potassium >5.1 mEq/L, and CKD stages 3‐4 were significantly associated with risk of CKD progression (*P* = .001, *P* = .03, *P* = .04, and *P* = .002, respectively; Table 2). Also, time between re‐examination visits was related to significant risk for outcome (HR, 0.97; 95% CI, 0.95‐0.99; *P =* .01). When these variables were included in the multivariate Cox regression analysis, FGF‐23 >528 pg/mL (HR, 11.33; 95% CI, 2.85‐44.98; *P* < .001), and potassium >5.1 mEq/L (HR, 12.74; 95% CI, 2.12‐76.73; *P* = .005) remained significantly associated with risk of CKD progression (Table 3).

## 4 DISCUSSION

Our study investigated the clinical relevance of increased FGF‐23 concentrations in dogs with CKD but without hyperphosphatemia. We found that increased serum FGF‐23 concentrations were significantly associated with risk of subsequent hyperphosphatemia and CKD progression in normophosphatemic dogs.

Various proposals for the pathophysiology of increased FGF‐23 concentrations in patients with CKD have been suggested.^20^, ^21^, ^22^ Of these mechanisms, we focused on the relationship between FGF‐23 and phosphate. Decreasing GFR in CKD decreases urinary phosphate excretion, resulting in phosphate accumulation. To compensate, circulating FGF‐23 concentrations increase to maintain phosphate homeostasis by enhanced urinary phosphate excretion.^11^, ^12^, ^13^ When GFR further decreases, decompensation of FGF‐23 occurs because of exacerbated phosphate excretory failure and decreased klotho protein, and patients with CKD develop hyperphosphatemia.^5^, ^13^, ^15^ In fact, increased blood FGF‐23 concentrations have been shown to occur earlier than hyperphosphatemia in humans, cats, and dogs with CKD.^16^, ^17^, ^18^, ^19^, ^23^ Therefore, we hypothesized that FGF‐23 would be predictive of the subsequent development of hyperphosphatemia in dogs with normophosphatemic CKD. In our study, increased serum FGF‐23 concentrations were significantly associated with the risk of developing subsequent hyperphosphatemia, suggesting that phosphate already may be accumulating in dogs with normophosphatemic CKD. Moreover, our results suggest that the period before phosphate accumulation becomes apparent as hyperphosphatemia was shorter in dogs with increased FGF‐23 concentrations. Another mechanism that contributes to increased blood FGF‐23 concentrations in CKD is decreased renal clearance itself because of decreased GFR.^20^ In humans, impaired GFR was the most important factor contributing to increased FGF‐23 concentrations in early stages of CKD.^35^ However, multivariate Cox regression analysis in our study showed that increased FGF‐23 remained a significant risk factor for development of hyperphosphatemia after adjusting for CKD stage (ie, magnitude of GFR). This finding suggests that phosphate accumulation in the body is an important factor involved in increased FGF‐23 concentrations, independent of GFR in dogs with CKD.

In addition, in our study, serum FGF‐23 concentration was significantly associated with the progression of CKD in dogs. In humans with CKD, increased FGF‐23 concentrations are related to shorter survival time.^24^, ^25^ One of the reasons for this phenomenon is that increased blood FGF‐23 concentrations increase the risk of cardiovascular comorbidities, such as myocardial infarction and heart failure in humans.^25^, ^36^, ^37^ In dogs and cats with CKD, increased plasma FGF‐23 concentrations also have been associated with shorter survival times.^9^, ^26^ However, myocardial infarction is a rare event in dogs and cats, and the relationship between FGF‐23 in CKD and heart failure has not been determined. Thus, the association between FGF‐23 and worse prognosis in dogs and cats with CKD cannot be explained by cardiovascular comorbidities. Another mechanism by which increased FGF‐23 may shorten survival time is that FGF‐23 is related to the progression of CKD in humans.^37^, ^38^, ^39^ Similar results also were observed in cats with CKD.^26^, ^40^ Our results are consistent with those of previous studies in humans and cats, but it is unknown whether FGF‐23 directly promotes CKD progression. In rats and beagles, phosphate loading was shown to lead to renal toxicity by calcification of the kidneys.^41^, ^42^, ^43^ Given the results of these studies, it is thought that the accumulation of phosphate in the body contributes to the progression of CKD by renal calcification. Therefore, increased blood FGF‐23 concentrations, which reflect phosphate accumulation, could be associated with progression of CKD and shorter survival time in dogs and cats with CKD. In previous studies, the prevalence of hyperphosphatemia in dogs with CKD was 0% to 18% in stage 1, 19% to 50% in stage 2, 50% to 90% in stage 3, and 100% in stage 4.^19^, ^44^, ^45^ Thus, the prevalence of hyperphosphatemia is associated with CKD stage and GFR.

Because hyperphosphatemia and the calcium × phosphorous product have been recognized as prognosis factors in dogs with CKD,^6^, ^7^, ^8^, ^9^, ^10^, ^46^, ^47^ phosphate restriction is an important treatment. For dogs, a renal diet consists of the restriction of protein, phosphate and salt, and the supplementation of omega‐3 fatty acids.^1^ Because various studies have indicated the benefits of a renal diet for dogs with CKD,^6^, ^7^, ^8^, ^48^, ^49^, ^50^ dietary modification has been commonly used to manage CKD in dogs. Of the nutrients in renal diets, phosphate is restricted to manage CKD‐MBD.^1^, ^5^ In fact, dietary phosphate and calcium restriction have been shown to prolong the survival of dogs in which functional renal mass was experimentally decreased by 15/16.^6^ Thus, phosphate restriction is thought to be a key treatment for phosphate accumulation in dogs with CKD. A previous study that showed the beneficial effect of a renal diet included dogs with spontaneous CKD and serum creatinine concentrations >2.0 mg/dL,^48^ and IRIS CKD guidelines recommend the initiation of a phosphate‐restricted diet beginning with stage 2.^30^ However, the efficacy of dietary management for dogs with CKD but without hyperphosphatemia has not been studied specifically. In addition, dogs with stage 2 CKD do not always have hyperphosphatemia.^19^, ^44^, ^45^ One study also has reported benefits of renal dietary management in dogs with stage 1 CKD.^51^ However, except for proteinuria, the application of dietary treatment for dogs with early‐stage CKD has not been established.^49^, ^52^ In humans and cats with CKD, because blood FGF‐23 concentrations are associated with survival time^24^, ^25^, ^26^ and decreased by feeding a renal diet,^53^, ^54^ FGF‐23 is recognized as a treatment target in patients with CKD. In our study, FGF‐23 was significantly associated with subsequent development of hyperphosphatemia, and their relationship remained significant even after adjusting for CKD stage. This result suggests that increased FGF‐23 concentrations can reflect a phosphate metabolic disturbance regardless of CKD stage in dogs. As such, phosphate restriction is expected to be effective for treating dogs with CKD that have abnormal phosphate metabolism. Therefore, FGF‐23 might be a useful marker to indicate the need for dietary treatment in dogs with CKD, especially those in stages 1 and 2. In addition, our findings suggest that decreasing serum FGF‐23 concentrations might be useful in preventing the development of hyperphosphatemia, but we cannot conclude that therapeutic intervention based on increased FGF‐23 concentration in dogs with CKD is beneficial because of the retrospective study design. In addition, in our study, no statistically significant difference was found between the presence and the absence of dietary treatment in the subsequent development of hyperphosphatemia and CKD progression. However, provision of a renal diet clearly affects mineral metabolism in dogs with CKD,^6^, ^7^ and we may not have observed these differences because of small sample size. Furthermore, because of the retrospective design, our study included dogs that were already receiving dietary management at baseline, which may have decreased their serum FGF‐23 concentrations. Our findings should be interpreted by considering that the diet of some of the dogs was modified during the study, and therefore the variable time exposure to dietary treatment could be an influencing factor. Thus, further prospective interventional studies are needed to determine the effect of dietary phosphate restriction on blood FGF‐23 concentrations, and whether dietary management for CKD dogs with normophosphatemia and increased FGF‐23 concentrations could prevent the development of hyperphosphatemia.

Our study could not establish a relationship between UPC or systemic hypertension and the progression of CKD, in contrast to previous studies.^33^, ^34^, ^55^ The reasons may include a difference in the study population, study design and therapeutic regimen. Although 23 of 42 dogs (55%) in our study were diagnosed as stage 1 CKD (serum creatinine concentration <1.45 mg/dL), previous studies targeted dogs with CKD having serum creatinine concentration >2.0 mg/dL.^33^, ^34^ Few studies have investigated factors associated with progression of early‐stage CKD in dogs, which can differ from those in more advanced stages. Moreover, our study was retrospective and included dogs that were already being treated for proteinuria, systemic hypertension or both, most of which were treated with telmisartan. The efficacy of telmisartan on proteinuria and blood pressure recently was found to be superior to that of angiotensin converting enzyme inhibitors.^56^, ^57^ Therefore, telmisartan could have influenced the effects of proteinuria and systemic hypertension on the progression of CKD.^33^, ^34^, ^55^

Our study had some limitations. First, the study was a retrospective investigation, and it may have been subject to selection bias. Some data sets had missing values, and the frequency of reassessment and therapeutic regimens such as dietary treatment and renin‐angiotensin system inhibitors were not standardized. Because time between re‐examination visits was significantly associated with the risk for outcomes, dogs with the development of hyperphosphatemia or CKD progression could be rigorously followed up and managed. In addition, follow‐up periods of some dogs with FGF‐23 <528 pg/mL and censored were not adequate to completely overcome these limitations. Second, sample size was small. Third, we did not consider the underlying causes of CKD in the dogs. Our study included various underlying diseases, such as protein‐losing nephropathy, renal dysplasia and other tubulointerstitial diseases. Because the progression rate of CKD and treatment strategies may differ based on the underlying disease, this factor could have affected the results of our study. Fourth, the upper limit of the reference range of FGF‐23 used in our study was determined using a small sample of healthy dogs.^19^ Therefore, a more precise cut‐off for the FGF‐23 concentration that is associated with the risk of the development of hyperphosphatemia and CKD progression is needed. Finally, our study did not consider the storage period of serum samples used for the measurement of FGF‐23 and intact PTH. Although storage stability in dogs was investigated for up to 28 days,^19^ the storage period of samples used in our study ranged from 3 months to 5 years. The storage period of the serum samples used may have affected the FGF‐23 and intact PTH concentrations obtained.

In conclusion, increased serum FGF‐23 concentration was a significant risk factor for the subsequent development of hyperphosphatemia in normophosphatemic dogs with CKD. In addition, serum FGF‐23 concentration was associated with CKD progression in the affected dogs. Future research that investigates whether a decrease in FGF‐23 concentrations prevents or delays the onset of hyperphosphatemia and associated progression is needed.

## CONFLICT OF INTEREST DECLARATION

Yuichi Miyagawa is in receipt of speaker honoraria from FUJIFILM VET Systems Co., Ltd. The other authors declare no potential conflict of interest.

## OFF‐LABEL ANTIMICROBIAL DECLARATION

Authors declare no off‐label use of antimicrobials.

## INSTITUTIONAL ANIMAL CARE AND USE COMMITTEE (IACUC) OR OTHER APPROVAL DECLARATION

Authors declare no IACUC or other approval was needed.

## HUMAN EHTICS APPROVAL DECLARATION

Authors declare human ethics approval was not needed for this study.

## References

[1] Polzin, DJ. Chronic kidney disease. In: Ettinger SJ, Feldman EC, Côté E: Text of Veterinary Internal Medicine. 8th ed St. Louis, MO: Elsevier, 2017:1938–1959.

[2] Hruska KA, Sugatani T, Agapova O, et al. The chronic kidney disease ‐ mineral bone disorder (CKD‐MBD): advances in pathophysiology. Bone. 2017;100:80‐86. 28119179 10.1016/j.bone.2017.01.023 PMC5502716

[3] Hou YC, Lu CL, Lu KC. Mineral bone disorders in chronic kidney disease. Nephrology (Carlton). 2018;23:88‐94. 30298663 10.1111/nep.13457

[4] Block GA, Klassen PS, Lazarus JM, et al. Mineral metabolism, mortality, and morbidity in maintenance hemodialysis. J Am Soc Nephrol. 2004;15:2208‐2218. 15284307 10.1097/01.ASN.0000133041.27682.A2

[5] Foster JD. Update on mineral and bone disorders in chronic kidney disease. Vet Clin North Am Small Anim Pract. 2016;46:1131‐1149. 27436330 10.1016/j.cvsm.2016.06.003

[6] Brown SA, Crowell WA, Barsanti JA, et al. Beneficial effects of dietary mineral restriction in dogs with marked reduction of functional renal mass. J Am Soc Nephrol. 1991;1:1169‐1179. 1768812 10.1681/ASN.V1101169

[7] Finco DR, Brown SA, Crowell WA, et al. Effects of phosphorus/calcium‐restricted and phosphorus/calcium‐replete 32% protein diets in dogs with chronic renal failure. Am J Vet Res. 1992;53:157‐163. 1539911

[8] Finco DR, Brown SA, Crowell WA, et al. Effects of dietary phosphorus and protein in dogs with chronic renal failure. Am J Vet Res. 1992;53:2264‐2271. 1476305

[9] Rudinsky AJ, Harjes LM, Byron J, et al. Factors associated with survival in dogs with chronic kidney disease. J Vet Intern Med. 2018;32:1977‐1982. 30325060 10.1111/jvim.15322 PMC6271312

[10] Pedrinelli V, Lima DM, Duarte CN, et al. Nutritional and laboratory parameters affect the survival of dogs with chronic kidney disease. PLoS One. 2020;15:e0234712. 32603378 10.1371/journal.pone.0234712 PMC7326232

[11] Fukagawa M, Kazama JJ. With or without the kidney: the role of FGF23 in CKD. Nephrol Dial Transplant. 2005;20:1295‐1298. 15840677 10.1093/ndt/gfh827

[12] Bär L, Stournaras C, Lang F, et al. Regulation of fibroblast growth factor 23 (FGF23) in health and disease. FEBS Lett. 2019;593:1879‐1900. 31199502 10.1002/1873-3468.13494

[13] Kovesdy CP, Quarles LD. Fibroblast growth factor‐23: what we know, what we don't know, and what we need to know. Nephrol Dial Transplant. 2013;28:2228‐2236. 23625971 10.1093/ndt/gft065 PMC3769978

[14] Shigematsu T, Kazama JJ, Yamashita T, et al. Possible involvement of circulating fibroblast growth factor 23 in the development of secondary hyperparathyroidism associated with renal insufficiency. Am J Kidney Dis. 2004;44:250‐256. 15264182 10.1053/j.ajkd.2004.04.029

[15] Evenepoel P, Meijers B, Viaene L, et al. Fibroblast growth factor‐23 in early chronic kidney disease: additional support in favor of a phosphate‐centric paradigm for the pathogenesis of secondary hyperparathyroidism. Clin J Am Soc Nephrol. 2010;5:1268‐1276. 20448073 10.2215/CJN.08241109 PMC2893066

[16] Geddes RF, Finch NC, Elliott J, et al. Fibroblast growth factor 23 in feline chronic kidney disease. J Vet Intern Med. 2013;27:234‐241. 23398216 10.1111/jvim.12044

[17] Isakova T, Wahl P, Vargas GS, et al. Fibroblast growth factor 23 is elevated before parathyroid hormone and phosphate in chronic kidney disease. Kidney Int. 2011;79:1370‐1378. 21389978 10.1038/ki.2011.47 PMC3134393

[18] Harjes LM, Parker VJ, Dembek K, et al. Fibroblast growth factor‐23 concentration in dogs with chronic kidney disease. J Vet Intern Med. 2017;31:784‐790. 28419560 10.1111/jvim.14707 PMC5435078

[19] Miyakawa H, Nagatani Y, Ogawa M, et al. Fibroblast growth factor‐23 as an early marker of CKD‐mineral bone disorder in dogs: preliminary investigation. J Small Anim Pract. 2020;61:744‐751. 33037651 10.1111/jsap.13244

[20] Razzaque MS. FGF23‐mediated regulation of systemic phosphate homeostasis: is klotho an essential player? Am J Physiol Renal Physiol. 2009;296:470‐476. 10.1152/ajprenal.90538.2008 PMC2660189 19019915

[21] Prié D, Torres PU, Friedlander G. Latest findings in phosphate homeostasis. Kidney Int. 2009;75:882‐889. 19190675 10.1038/ki.2008.643

[22] Rodelo‐Haad C, Santamaria R, Muñoz‐Castañeda JR, et al. FGF23, biomarker or target? Toxins (Basel). 2019;11:175. 10.3390/toxins11030175 PMC6468608 30909513

[23] Gutierrez O, Isakova T, Rhee E, et al. Fibroblast growth factor‐23 mitigates hyperphosphatemia but accentuates calcitriol deficiency in chronic kidney disease. J Am Soc Nephrol. 2005;16:2205‐2215. 15917335 10.1681/ASN.2005010052

[24] Isakova T, Xie H, Yang W, et al. Fibroblast growth factor 23 and risks of mortality and end‐stage renal disease in patients with chronic kidney disease. JAMA. 2011;305:2432‐2439. 21673295 10.1001/jama.2011.826 PMC3124770

[25] Seiler S, Reichart B, Roth D, et al. FGF‐23 and future cardiovascular events in patients with chronic kidney disease before initiation of dialysis treatment. Nephrol Dial Transplant. 2010;25:3983‐3989. 20525642 10.1093/ndt/gfq309

[26] Geddes RF, Elliott J, Syme HM. Relationship between plasma fibroblast growth factor‐23 concentration and survival time in cats with chronic kidney disease. J Vet Intern Med. 2015;29:1494‐1501. 26403212 10.1111/jvim.13625 PMC4895675

[27] International Renal Interest Society . 2019. http://www.iris-kidney.com/pdf/IRIS_Staging_of_CKD_modified_2019.pdf. Accessed February 1, 2021.

[28] Acierno MJ, Brown S, Coleman AE, et al. ACVIM consensus statement: guidelines for the identification, evaluation, and management of systemic hypertension in dogs and cats. J Vet Intern Med. 2018;32:1803‐1822. 30353952 10.1111/jvim.15331 PMC6271319

[29] Kanda Y. Investigation of the freely available easy‐to‐use software “EZR” for medical statistics. Bone Marrow Transplant. 2013;48:452‐458. 23208313 10.1038/bmt.2012.244 PMC3590441

[30] International Renal Interest Society . 2019. http://www.iris-kidney.com/pdf/IRIS-DOG-Treatment_Recommendations_2019.pdf. Accessed February 1, 2021.

[31] Johnson RA. A quick reference on respiratory acidosis. Vet Clin North Am Small Anim Pract. 2017;47:185‐189. 27939862 10.1016/j.cvsm.2016.10.012

[32] de Brito Galvão JF, Schenck PA, Chew DJ. A quick reference on hypercalcemia. Vet Clin North Am Small Anim Pract. 2017;47:241‐248. 28012787 10.1016/j.cvsm.2016.10.016

[33] Jacob F, Polzin DJ, Osborne CA, et al. Evaluation of the association between initial proteinuria and morbidity rate or death in dogs with naturally occurring chronic renal failure. J Am Vet Med Assoc. 2005;226:393‐400. 15702689 10.2460/javma.2005.226.393

[34] Jacob F, Polzin DJ, Osborne CA, et al. Association between initial systolic blood pressure and risk of developing a uremic crisis or of dying in dogs with chronic renal failure. J Am Vet Med Assoc. 2003;222:322‐329. 12564594 10.2460/javma.2003.222.322

[35] Filler G, Liu D, Huang SH, et al. Impaired GFR is the most important determinant for FGF‐23 increase in chronic kidney disease. Clin Biochem. 2011;44:435‐437. 21291879 10.1016/j.clinbiochem.2011.01.009

[36] Kendrick J, Cheung AK, Kaufman JS, et al. FGF‐23 associates with death, cardiovascular events, and initiation of chronic dialysis. J Am Soc Nephrol. 2011;22:1913‐1922. 21903574 10.1681/ASN.2010121224 PMC3187186

[37] Kuczera P, Adamczak M, Wiecek A. Fibroblast growth factor‐23‐a potential uremic toxin. Toxins (Basel). 2016;8:369. 10.3390/toxins8120369 PMC5198563 27941640

[38] Fliser D, Kollerits B, Neyer U, et al. Fibroblast growth factor 23 (FGF23) predicts progression of chronic kidney disease: the mild to moderate kidney disease (MMKD) study. J Am Soc Nephrol. 2007;18:2600‐2608. 17656479 10.1681/ASN.2006080936

[39] Portale AA, Wolf MS, Messinger S, et al. Fibroblast growth factor 23 and risk of CKD progression in children. Clin J Am Soc Nephrol. 2016;11:1989‐1998. 27561289 10.2215/CJN.02110216 PMC5108188

[40] Liao Y, Chou C, Lee Y. The association of indoxyl sulfate with fibroblast growth factor‐23 in cats with chronic kidney disease. J Vet Intern Med. 2019;33:686‐693. 30779214 10.1111/jvim.15457 PMC6430881

[41] Mackay EM, Oliver J. Renal damage following the ingestion of a diet containing an excess of inorganic phosphate. J Exp Med. 1935;61:319‐334. 19870361 10.1084/jem.61.3.319 PMC2133223

[42] Haut LL, Alfrey AC, Guggenheim S, et al. Renal toxicity of phosphate in rats. Kidney Int. 1980;17:722‐731. 7412107 10.1038/ki.1980.85

[43] Schneider P, Pappritz G, Müller‐Peddinghaus R. Potassium hydrogen phosphate induced nephropathy in the dog. I. Pathogenesis of tubular atrophy (author's transl). Vet Pathol. 1980;17:699‐719. 7423830 10.1177/030098588001700607

[44] Cortadellas O, Fernández del Palacio MJ, Talavera J, et al. Serum phosphorus concentrations in dogs with leishmaniosis at different stages of chronic kidney disease. Vet Rec. 2009;164:487‐490. 19377087 10.1136/vr.164.16.487

[45] Cortadellas O, Fernández del Palacio MJ, Talavera J, et al. Calcium and phosphorus homeostasis in dogs with spontaneous chronic kidney disease at different stages of severity. J Vet Intern Med. 2010;24:73‐79. 19925576 10.1111/j.1939-1676.2009.0415.x

[46] Lippi I, Guidi G, Marchetti V, et al. Prognostic role of the product of serum calcium and phosphorus concentrations in dogs with chronic kidney disease: 31 cases (2008‐2010). J Am Vet Med Assoc. 2014;245:1135‐1140. 25356714 10.2460/javma.245.10.1135

[47] Lucero MC, Duque FJ, Gil M, et al. A plasma calcium‐phosphorus product can be used to predict the lifespan of dogs with chronic kidney disease. Can Vet J. 2019;60:1319‐1325. 31814639 PMC6855231

[48] Jacob F, Polzin DJ, Osborne CA, et al. Clinical evaluation of dietary modification for treatment of spontaneous chronic renal failure in dogs. J Am Vet Med Assoc. 2002;220:1163‐1170. 11990962 10.2460/javma.2002.220.1163

[49] Cortadellas O, Talavera J, Fernández del Palacio MJ. Evaluation of the effects of a therapeutic renal diet to control proteinuria in proteinuric non‐azotemic dogs treated with benazepril. J Vet Intern Med. 2014;28:30‐37. 24372810 10.1111/jvim.12246 PMC4895532

[50] Zatelli A, Roura X, D'Ippolito P, et al. The effect of renal diet in association with enalapril or benazepril on proteinuria in dogs with proteinuric chronic kidney disease. Open Vet J. 2016;6:121‐127. 27540513 10.4314/ovj.v6i2.8 PMC4980477

[51] Hall JA, Fritsch DA, Yerramilli M, et al. A longitudinal study on the acceptance and effects of a therapeutic renal food in pet dogs with IRIS‐stage 1 chronic kidney disease. J Anim Physiol Anim Nutr (Berl). 2018;102:297‐307. 28276099 10.1111/jpn.12692

[52] IRIS Canine GN Study Group Standard Therapy Subgroup , Brown S, Elliott J, Francey T, et al. Consensus recommendations for standard therapy of glomerular disease in dogs. J Vet Intern Med. 2013;27:S27‐S43. 24635378 10.1111/jvim.12230

[53] Goto S, Nakai K, Kono K, et al. Dietary phosphorus restriction by a standard low‐protein diet decreased serum fibroblast growth factor 23 levels in patients with early and advanced stage chronic kidney disease. Clin Exp Nephrol. 2014;18:925‐931. 24578219 10.1007/s10157-014-0947-4

[54] Geddes RF, Elliott J, Syme HM. The effect of feeding a renal diet on plasma fibroblast growth factor 23 concentrations in cats with stable azotemic chronic kidney disease. J Vet Intern Med. 2013;27:1354‐1361. 24010686 10.1111/jvim.12187

[55] Wehner A, Hartmann K, Hirschberger J. Associations between proteinuria, systemic hypertension and glomerular filtration rate in dogs with renal and non‐renal diseases. Vet Rec. 2008;162:141‐147. 18245745 10.1136/vr.162.5.141

[56] Lourenço BN, Coleman AE, Brown SA, et al. Efficacy of telmisartan for the treatment of persistent renal proteinuria in dogs: a double‐masked, randomized clinical trial. J Vet Intern Med. 2020;34:2478‐2496. 33165969 10.1111/jvim.15958 PMC7694823

[57] Fowler BL, Stefanovski D, Hess RS, et al. Effect of telmisartan, angiotensin‐converting enzyme inhibition, or both, on proteinuria and blood pressure in dogs. J Vet Intern Med. 2021;35:1231‐1237. 10.1111/jvim.16102 33769606 PMC8163128

